# Combining learning for educators and participants in a paediatric CPD programme

**DOI:** 10.1186/s12909-019-1461-x

**Published:** 2019-01-21

**Authors:** Daniel Holmgren, Maria Skyvell-Nilsson, Per Wekell

**Affiliations:** 1 Department of Pediatrics, Skaraborg Hospital, Skövde, Sweden; 2 Department of Health Sciences, University West, Trollhättan, Sweden; 3 Department of Pediatrics, NU-Hospital Group, Uddevalla, Sweden; 4 Institute of Clinical Sciences, University of Gothenburg, Gothenburg, Sweden

## Abstract

**Background:**

Most continuing professional development (CPD) programmes do not include an educational training module. In our country, educational practice in the areas of CPD and continuing medical education relies traditionally on conventional lectures. This is in sharp contrast to the educational research that clearly demonstrates that educational programmes emphasising adult learning methods have greater potential to change physicians’ clinical practice. To investigate whether lecture-oriented educators were prepared to change their educational practice towards principles of adult learning, we decided to combine learning for educators and participants in a paediatric CPD programme.

The aim of the study was to investigate educators’ reflections on their learning and educational practice after they have undergone an educational skills component integrated in the implementation of a CPD learning module for paediatricians and evaluate the results from the participants’ perspective.

**Methods:**

The objectives of the educational skills component of the learning module were developed according to adult learning theories. The learning objectives for the CPD learning module were based on a pre-course needs assessment. Evaluations were made using questionnaires.

**Results:**

Seven of 10 participants in the educational skills component of the learning module and all the participants, 13 paediatricians and 14 nurses, who participated in the learning module, answered the questionnaires.

The results of this pilot study show that educators whose main experience of teaching was based on lectures were strengthened in their practice; they defined their competence and were prepared to move towards adult learning principles. The participants in the learning module expressed a high degree of satisfaction.

**Conclusions:**

We conclude that it is feasible to combine learning for educators and participants in a paediatric CPD programme and that lecture-oriented educators are prepared to change their educational practice towards principles of adult learning.

**Electronic supplementary material:**

The online version of this article (10.1186/s12909-019-1461-x) contains supplementary material, which is available to authorized users.

## Background

The importance of providing physicians with continuing professional development (CPD) has been emphasised in Europe in recent years and the EU Professional Qualifications Directive has imposed increased requirements for CPD on EU member states [1]. In Sweden, the sponsorship of physicians’ CPD by the pharmaceutical industry has gradually been restricted and there has been a growing national consensus that CPD for physicians has to be reinforced [2]. In our country, educational practice in the areas of CPD and continuing medical education relies traditionally on conventional lectures. This is in sharp contrast to the educational research that clearly demonstrates that educational programmes emphasising adult learning methods have greater potential to change physicians’ clinical [3].

As Directors of Studies, we (DH, PW) have a mandate to provide CPD programmes for paediatricians in western Sweden, a region with 1.6 million inhabitants, including 320,000 children below the age of 18. In 2010, we introduced a coherent two-year CPD programme for on-call consultant paediatricians, which was developed from a needs assessment in the target group [4, 5]. The learning process for each learning module in the programme was based on three steps: 1) *preparation*, including reading assignments, 2) *application of knowledge*, for example, case discussions, and 3) *assessments* such as written, group-based home examination. During the implementation of the programme, we found that the participants’ diverse experience and expertise provided a strong interactive learning environment, in which the participants often had the capacity to take on the role of formal educators in their area of expertise [4, 6]. The participants in this programme made it very clear that they preferred adult learning principles to traditional lectures, both as participants and as educators (DH, PW 2014, unpublished data). Based on the experience from the first CPD programme, we wanted to promote the practice of adult learning methods in addition to paediatric competence in a second coherent CPD programme targeting paediatricians working with general paediatrics at outpatient clinics.

The idea of integrating an educational skills component was piloted in the first learning module of the CPD programme for outpatient paediatricians, a module that addressed refugee children’s problems under the heading: “Refugee children – focusing on health examinations”. This module was necessitated by the large influx of refugee children in Sweden in 2015 [7] that exposed a need to strengthen health investigations in western Sweden. The importance of the module was also highlighted in a pre-course needs assessment carried out as part of the curriculum development of the CPD programme. The pre-course needs assessment was completed using a questionnaire targeting all outpatient paediatricians in the region. A total of 46 paediatricians, 33 women and 13 men, participated in the needs assessment, corresponding to 75% of the target group and consistent with the gender ratio in that group. A total of 355 clinical situations or problems were proposed. Categories and themes were extracted by qualitative content analysis of the survey results [8] and informed the development of overall objectives, as well as specific objectives for each learning module. A total of 19 learning modules were drafted based on the needs assessment, of which *Refugee children – focusing on health examinations* was the first learning module to be implemented. As health examination is largely a task for teams of paediatric nurses and paediatricians, paediatric nurses were also invited to participate in this specific CPD learning module.

The learning in the learning module was based on a constructivist view of learning, where learning is the process of constructing new knowledge on the foundation of what you already know using educational principles of adult learning, such as the importance of relevance, different learning styles, goal- and problem-oriented learning and taking advantage of participants’ knowledge and experience in learning activities [3].

The curriculum of the learning module and the implementation of the learning activities were not derived from one specific learning theory. Nevertheless, there was a basic learning approach throughout the programme that correlates to instrumental learning theories, emphasising the development of competence and the training of skills in specific clinical situations [3]. Furthermore, one reason for making the whole CPD programme coherent was that the participants should have time to get to know each other, discuss and reflect on their learning, in order to find the courage to discover what they did not even know that they did not know. An educational aim of this kind may relate to transformative learning theories and reflective learning models [3]. Finally, the learning of educators together with the development of collegial networks have the potential to influence the learning environment in the whole region, which could be seen as an educational approach based on social learning theories [3].

The planning and evaluation within the curriculum was inspired by Miller’s pyramid, which meant that the learning outcomes for both the educators and the participants should be associated with verbs, rather than lists of things to learn [3, 9]. In the pre-course planning, it become clear that the educators’ teaching experience was mainly based on lectures. We therefore decided to investigate whether the educators were prepared to change their educational practice and use adult learning methods.

### Aim of the study

To investigate educators’ reflections on their learning and educational practice after they have completed an educational skills component integrated in the implementation of a CPD learning module for paediatricians and evaluate the results of their educational practice during the programme from the participants’ perspective.

## Methods

Participants in the educational skills component were the educators in the CPD learning module entitled “Health examination of refugee children” and they will hereafter be referred to as “the educators” in the present study. The educators were recruited from among key experts in the region and from the participants in the CPD learning module with expertise in the field. Specifically, the educators consisted of two nurses, two infectious-disease specialists and six paediatricians.

The participants in the CPD learning module consisted of 13 paediatricians and 14 paediatric nurses and will henceforth be referred to as “the participants”. The vast majority of the participating paediatricians had many years of professional experience and CPD courses. Most participating nurses had long experience of working with child health and paediatric services in an outpatient setting. Their educational training varied, but, as far as we know, none of the participants had any major experience or formal education in directing case discussions.

The Directors of Studies were responsible for the framework of the learning module, including the educational skills component, which meant formulating the learning strategy, defining the elements in the educational skills component and supervising the educators participating in the educational skills component, as well as developing the structure and overall objectives of the CPD learning module.

### The educational skills component

The implementation of the educational skills component stretched over a period of about six to 10 weeks and began with the Directors of Studies introducing the CPD learning module in general and the educational skills component for the educators in particular.

All the educators were specialists in their respective area and had a solid competence base. For the majority, this meant that they had been working and teaching in their field for more than 10 years. None of the educators had any substantial experience of or training in directing case discussions. After the introduction, the educators and the Directors of Studies communicated by e-mail, Skype and physical meetings. Meanwhile, the educators worked on their own or together, without the direct involvement of the Directors of Studies. The educational skills component was incorporated into, completed and evaluated during the implementation and evaluation of the CPD learning module.

#### Learning objectives

The learning objectives for the educational skills component were adapted to the anticipated learning outcomes of the learning module and consequently formulated in terms of verbs in the following way: *“After participating in the educational skills component, you should be able to: I) Assess the learning needs of the participants in a learning module, II) develop clear and relevant learning objectives for a learning module, III) prepare reading assignments according to the objectives, IV) prepare cases according to the objectives, including key learning points, and V) facilitate case discussions*” [10].

#### Learning process

The learning in the educational skills component covered five areas corresponding to the objectives.

##### I. Assessing learning needs

The educators assessed the learning needs of the participants in the learning module by examining the categories and sub-categories that related to the health examination of refugee children in the above-mentioned needs assessment. The result of the needs assessment then informed the drafting of the overall and specific objectives for the learning module.

##### II. Developing learning objectives

The learning objectives for the learning module were developed by the educators and the Directors of Studies in close collaboration and were further refined according to the expertise of the educators. The objectives were finally formulated in general terms: “*After completing the learning module, you should be able to* … ,” as shown in Additional file 1: Table S1, following a recommended protocol by Aspegren 2007 [10].

##### III. Preparing reading assignments

Reading assignments were prepared by the educators in close collaboration with the Directors of Studies and distributed to the participants 3 weeks before the learning module was implemented.

##### IV. Preparing cases

The educators prepared cases according to the learning objectives for the learning module, including key learning points, under the supervision of the Directors of Studies.

##### V. Implementing the course

A two-day programme was created; it began with half a day of traditional lectures for problem orientation with the following titles *Disease panorama in the country of origin*, *Vaccinations*, *Meeting refugee children*, *Health and illness in refugee children* and *Legislation and regulations for health care in refugee children*. After the lectures, one and a half days were dedicated to case discussions that were conducted by the educators together with the Directors of Studies. The case discussions were held in three parallel sessions, with the following headings: skin diseases, blood-borne diseases, immunisation, mental health problems, tuberculosis and other diseases.

During the implementation of the learning module, the educators facilitated case discussions in pairs, together with one of the Directors of Studies, in two thirds of the sessions. This meant that the educators were able to practise how to facilitate case discussions with the same content/cases but with different groups of participants on at least three occasions, which gave them the opportunity to experiment with a new approach to learning, while obtaining informal feedback from their fellow educators and the Directors of Studies. The case discussions were conducted with the participants seated in groups of four to five. The educators presented the cases with clinical problems corresponding to the objectives of the learning module. The participants worked in their groups to solve the problems. The discussion was then opened up in the larger group by the educators, starting by getting one of the groups to explain how they would address the problem. At this point, discussions between the groups were encouraged by the educators. Finally, the case was summarised by the educators in terms of key learning points, or by the participants under the supervision of the educators.

#### Evaluation

The educational skills component was evaluated in four ways: 1) by the participants’ satisfaction with the CPD learning module and the extent to which they were prepared to recommend it to a colleague, 2) by the participants’ assessment of the CPD learning module’s strengths and weaknesses, 3) by assessing the participants’ reflections on their learning and clinical practice; these three evaluations were made immediately after the completion of the learning module and 4) by assessing the educators’ reflections on their learning and educational practice, one to 2 weeks later. More specifically, the evaluation questionnaires are illustrated in Additional file 2: Table S2.

The questions regarding the participants’ satisfaction with the CPD learning module and the degree to which they would recommend it to colleagues, as well as the open questions with respect to the module’s three strengths and weaknesses, have been used for more than 10 years for the evaluation of CPD courses for paediatricians in the region. They were originally derived from a questionnaire used for the evaluation of courses for doctors under specialist training [11].

The open questions on the participants’ and educators’ reflections on their learning were formulated by the authors, informed by *The role of reflection in CPD and revalidation* by Liu and Brennan 2012 [12].

The time interval between the different evaluations meant that the educators had been informed of the results of the participants’ assessment of satisfaction with the CPD learning module when they answered the questions about their intended change in educational practice.

All the answers to the open questions, from both the educators and the participants, were analysed by qualitative content analysis [8]. The analysis was made by all the authors in three steps: *extracting condensed meaning units*, *categorisation of data* and *deriving sub-themes and themes*, separately for educators and participants, as well as for physicians and nurses.

## Results

### Participants’ satisfaction with the CPD learning module

All the participants, 13 paediatricians and 14 paediatric nurses, answered the questionnaires.

The median response (on a six-point scale) to the questions relating to how they evaluated the learning module and whether they would recommend the learning module was 5.8 and 5.9 respectively.

### Participants’ assessment of the strengths and weaknesses of the CPD learning module

All the responses to the question relating to the strengths of the CPD learning module belonged to and could be sorted under the headings of the specific learning objectives of the educational skills component, illustrated in Additional file 3: Table S3.

A total of 24 items were mentioned in the answers to the question relating to the weaknesses of the CPD learning module of which 14 answers stated that there were no weaknesses. The categorisation of the rest of the answers is illustrated in a footnote in Additional file 3: Table S3.

### Participants’ reflections on their learning and clinical practice

From the participants’ answers to the questions on learning and clinical practice, one main theme, “*Improvement in clinical practice”,* and four subthemes from three perspectives emerged. The responses were analysed separately for physicians and nurses, but, as the analysis revealed the same major themes and subthemes in both groups, they are presented together, as shown in Additional file 4: Table S4.

The participants themselves stated that they considered their learning important in their clinical practice, but they were also aware of the limitations in their competence, as well as the need to learn more and how to achieve this. As one of the paediatricians who participated in the learning module suggested, “*Individual study. Consulting colleagues mostly when I have tricky cases. Auscultation, at a tuberculosis clinic, for example”.* They also indicated that they had been alerted to the vulnerability of refugee children, emphasising the importance of acting in the interests of the child and seeing the child as a whole. As one participant expressed it, *“I will ask more about life as a whole. Who is the child’s trustee? Do they have contact? Does it work well? If this does not work, I know that I will contact the legal representative/Migration Board. Contact social services if accommodation is not good”*.

They further highlighted the importance of understanding and developing their professional roles and improving collaboration, which can be seen as a response to a new health problem – the health problems of refugee children – in the increasingly specialised and complex health-care context in which they work. This is illustrated by one of the participants’ answers: *“Be better at identifying mental health problems. The problem is that it is difficult to know who to turn to for support – the school? Psychologist at the paediatric outpatient clinic? Child psychiatry clinic? This is not obvious in our municipality”*.

### Educators’ reflections on their learning and educational practice

Seven of 10 educators answered the questionnaire. They all replied that their experience as an educator during the programme will change their way of teaching in the future and all but one said that they had been strengthened in their role as an educator.

Again, these themes were the same for paediatricians and nurses and they are therefore presented together, see Additional file 5: Table S5.

Nearly all the educators who responded to the survey said that their participation in the educational skills component of the learning module had supported them in their role as educators and, at the same time, all of them were prepared to make changes in their educational practice. As one of the educators put it, *“It has become even clearer that regular lectures are outdated, ineffective and educationally inferior all round”*. There was a clear and strong direction in the intention to make changes in educational practice, towards a willingness to practise adult learning methods; for example, one of the educators said: *“I very much appreciated the problem-based approach, the active search for knowledge and creative discussions in the group. Yes, this course made me more interested in this kind of educational activity”.*

The evaluation also showed that the educators had understood the importance of relevance and the participants’ contribution to the learning, which is illustrated in the following two quotations: *“The fact that the course is based on a needs assessment creates the right focus and dedication among the participants”* and *“Case discussions give the participants motivation and the fact that they make use of each other’s competence means that their precise situation, the reality they work in, is elucidated the most”*. The educators were also prepared to re-evaluate their own approach to their role as educators, as judged by the answers from two of the educators to the question about how to address their intended changes in educational practice: *“To be more of a guide than a ‘superior lecturer’”* and *“To listen more ... and talk less*”.

## Discussion

Health care for children in Sweden faces a number of challenges to preserve their good health [7, 13–15]. The present CPD learning module was developed and implemented in response to a new situation in which the number of asylum-seeking minors tripled between 2014 and 2015 in Sweden, reaching 70,000 in 2015, including 35,000 unaccompanied minors [7]. The relevance of the learning module was ensured by allowing the clinical situations and problems the participants face in everyday practice to frame the curriculum formulation and the learning process [16, 17].

The present pilot study is an attempt formally to combine learning for educators and participants in a CPD programme. In this first step, we chose to direct our educational efforts at the educators per se. The results show that educators whose main experience of teaching is based on lectures are prepared to change their educational practice and apply adult learning principles.

In the educational skills component, the educators participated in the design of the different parts of the learning module and practised a range of educational skills, from the formulation of learning objectives to the facilitation of case discussions. They received direct feedback from the Directors of Studies, throughout the whole process, and from their fellow educators and participants during the case discussions. The educators were therefore able to reflect on their own learning and their educational practice, described as “reflection-in-action” by Schön [18].

During the learning module, it was noted that the participants had a positive attitude towards the educators’ efforts, in keeping with the results of the participants’ evaluation of satisfaction, strengths and weaknesses. The participants’ positive attitude may thus have stimulated the educators’ interest in adult learning methods and their willingness to change their educational practice. The educators evaluated their learning one to 2 weeks after the completion of the learning module, which meant that they had time to reflect on what they had experienced and accomplished before they answered the questions on intended change in educational practice, as depicted by Taylor et al., “Reflection on action” [3]. Even if the educators said that they were prepared to change their educational practice towards adult learning principles, it remains to be seen whether their new knowledge changed them in depth [19] and whether they will use their new experience in their forthcoming educational practice.

A faculty development programme like the educational skills component in the present study is theoretically designed to strive to reach the top of Miller’s pyramid – “Doing” [9]. This is made most obvious by the educators facilitating case discussions, where the educators’ “doing” is evaluated by the participants and by the educators’ self-assessment. The analyses of data from the evaluation of strengths and weaknesses emphasised the different elements of the educational skills component, in which the educators played an active part in assessing learning needs, developing learning objectives, preparing reading assignments and formulating cases. The educators’ “doing” was thus integrated in the curriculum formulation process that preceded the implementation of the learning module.

The participants’ evaluation of the learning module shows that the educational skills component was implemented and integrated with high scores for overall satisfaction and willingness to recommend it to colleagues. The majority of participants reported that they had been strengthened in their clinical practice, but also that they were prepared to make changes in their practice, corresponding to Level 2, or “Learning”, in Kirkpatrick’s model for the evaluation of training programmes [20].

During the learning module, we observed a learning environment in which experienced physicians and nurses shared their knowledge and competence, learned from one another, created networks and developed their teamwork. The learning environment also appeared to create the comfort zone needed for both educators and participants to have the courage to identify areas that they needed to strengthen and try new methods in educational practice, as well as discussing their clinical practice. A learning environment of this kind is similar to a type of “Community of practice” [21] and has the potential to influence learning environments in the region when the participants return to their clinical, educational and academic duties [22].

After the educators had completed the educational skills component of our programme, their reflections and the participants’ evaluation showed that they are both inclined and competent to apply and implement educational initiatives based on adult learning principles. This corresponds to the highest levels, levels 3 (Apply) and 4 (Targeted outcome), in Kirkpatrick’s model for the evaluation of training programmes [20]. The result has encouraged us to take our educational efforts one step further, by introducing an educational skills module directed at all participants in the forthcoming paediatric CPD programme. The evaluation of the effect of an effort of this kind on the learning environment, the level of knowledge among colleagues and the impact on children’s well-being and health in the region will be a challenge for future research.

### Methodological considerations

The questionnaires used in the present study have not been validated against any established instrument within the area of interest. On the other hand, the questions about participants’ satisfaction and assessment of the strengths and weaknesses of the CPD learning module have been successfully used within the paediatric CPD programme for more than 10 years. The questions about educators’ and participants’ reflections on learning and change in practice were adapted from *The role of reflection in CPD and revalidation* for anaesthetists by Liu & Brennan 2012 [12].

The number of educators that took part in this pilot study was limited and not all the educators answered the questionnaire. However, the themes and subthemes in the qualitative content analysis were strong and we do not believe that the results would have been significantly different if the number of educators had been higher. As the number of educators was limited and we included all the available data in the analyses, the concept of saturation is not applicable to this study, but it is worth noting that new themes did not appear at the end of the analyses. We think that the transferability of our findings is increased by the wide variety of the educators’ academic level and educational experience but also by what educators have expressed in previous CPD programmes with the same educational strategy and principles. The follow-up period between the implementation and the evaluation of the educational skills component was short and we do not know whether the educators will use what they learned in the future. As a result, we intend to follow the way educators change their educational practice over time.

## Conclusions

The results of this pilot study show a high degree of satisfaction with the learning module among the participants; both educators and participants were strengthened in their respective practice and were prepared to define their competence, make changes in their practice and learn more. We conclude that the results of the study indicate that it is feasible to combine learning for educators and participants in a paediatric CPD programme and that lecture-oriented educators are prepared to change their educational practice towards principles of adult learning in our setting. The results of the present study therefore encourage us to introduce educational skills components in future CPD programmes as the next step.

## Additional files

Additional file 1: **Table S1**. Final formulation of the specific objectives for the learning module (DOC 44 kb)

Additional file 2: **Table S2**. The evaluation questionnaires (DOCX 23 kb)

Additional file 3: **Table S3**. The responses from 27 participants to the question: “*Which were the three main strengths of the learning module?”* categorised and sorted under the headings of the specific learning objectives of the educational skills component (DOC 177 kb)

Additional file 4: **Table S4**. Participants’ reflections in responses to the open questions on learning and clinical practice: major theme, themes and subthemes (DOC 51 kb)

Additional file 5: **Table S5**. Educators’ reflections in response to the open questions on learning and educational practice: major theme, themes and subthemes (DOC 48 kb)

## Abbreviations

CPD: continuing professional development
