# Supplementary material for: Combining learning for educators and participants in a paediatric CPD programme
Source: BMC Med Educ. 2019 Jan 21;19:28. doi: 10.1186/s12909-019-1461-x (PMC6341706; doi:10.1186/s12909-019-1461-x)
Supplement: Supplementary file 1 — Table S1. Final formulation of the specific objectives for the learning module (DOC 44 kb) [file 12909_2019_1461_MOESM1_ESM.doc]

**Table 1. Final formulation of the specific objectives for the learning module**

**Aim** The learning module *Refugee children – focusing on health investigations* is part of a two-year CPD programme for paediatricians at outpatient clinics, with the aim of enabling the participants to develop the competence required to function as a senior general paediatrician at an outpatient clinic.

**Objective**: After completing the learning module, participants should be able to conduct a health examination of newly arrived refugee children and adolescents by:

***Knowing***

**Certain circumstances that affect the situation of the child**, such as:

Conditions in the country of origin

- Disease panorama
- Health care – especially vaccinations
- National and international conflicts
- Culture

Vulnerability during the escape

Conditions in the new country (Sweden)

- The asylum process
- Human rights, including the UN Convention on the Rights of the Child and the status of refugees
- Health investigations

**The information to be included in the offer of** **a health examination:**

- Purpose
- Voluntary nature
- Right to an interpreter
- Contact information to the health-care provider conducting the health investigation

***Being able to***

**Perform a relevant health investigation** including:

- Medical history
- Vaccination history
- Physical examination
- Relevant investigations
- Information about health and dental care

**Assess the results of the health investigation** with special focus on:

- Vaccination status
- Blood-borne diseases – hepatitis B and C, HIV
- Tuberculosis – latent and active tuberculosis
- Skin diseases and symptoms – scabies, impetigo, diphtheria and leishmaniosis
- MRSA carriership
- Mental problems and illness

**Draw up a health plan based on the results of the health examination and implement the plan** by:

- Referring relevant problems to paediatric, child health and/or school health services for continued care
- Carrying out relevant parts of the plan at your outpatient clinic
- Referring the child to relevant investigations and specialists
- Following up and informing the child of the results of the health investigation
- Following up your own and referred patients who fail to attend planned visits and investigations
