# Supplementary material for: Combining learning for educators and participants in a paediatric CPD programme
Source: BMC Med Educ. 2019 Jan 21;19:28. doi: 10.1186/s12909-019-1461-x (PMC6341706; doi:10.1186/s12909-019-1461-x)
Supplement: Supplementary file 2 — Table S2. The evaluation questionnaires (DOCX 23 kb) [file 12909_2019_1461_MOESM2_ESM.docx]

**Table 2.** **The evaluation questionnaires**

***Participants’ satisfaction with the CPD learning module***

1) *“How would you evaluate the learning module in general?”* (rating scale 1-6, where 1 = very bad and 6 = very good) and 2) *“Would you recommend the learning module to a colleague in the same position as yours?”* (rating scale 1-6, where 1 = not at all and 6 = definitely).

***Participants’ assessment of strengths and weaknesses of the CPD learning module***

1) *“Which were the three main strengths of the learning module?”* and 2) “*Which were the three main weaknesses of the learning module?”*

***Participants’ ~~re~~flection on their learning and clinical practice***

*1) “Will the present learning module change your way of implementing health examinations?* *If so, in which way?”*

2) *Has the present learning module strengthened you in the way you implement health examinations? If so, in which areas?”* 3) *“Has the present learning module identified anything more you need to learn or develop?”* and 4) *“How could you deal with that?”*.

***Educators’ reflection on their learning and educational practice***

1) “*Will your experience as an educator during the programme change your way of teaching in the future? If so, in what way?*” 2) “*Has your experience as an educator during the programme strengthened you in your role as an educator? If so, in what way?*” 3) “*Has your experience as an educator during the programme focused your attention on anything further you need to learn or develop as an educator*?” and 4) “*If you have experienced something you need to learn or develop as an educator, how could you deal with that*?”.
