# Supplementary material for: Combining learning for educators and participants in a paediatric CPD programme
Source: BMC Med Educ. 2019 Jan 21;19:28. doi: 10.1186/s12909-019-1461-x (PMC6341706; doi:10.1186/s12909-019-1461-x)
Supplement: Supplementary file 3 — Table S3. The responses from 27 participants to the question: “Which were the three main strengths of the learning module?” categorised and sorted under the headings of the specific learning objectives of the educational skills component (DOC 177 kb) [file 12909_2019_1461_MOESM3_ESM.doc]

**Table 3. The responses from 27 participants to the question: “*Which were the three main strengths of the learning module?”* categorised and sorted under the headings of the specific learning objectives of the educational skills component**

- **Identify the skills the participants in a learning module need Items (n)**

Category **Relevant needs** 7

Quotes: *“Reality based”*

*“Very educational, practical and applicable.*

*Applies to patients I could meet”*

*“Good to participate in the course together with*

*nurses in the same team”*

- **Develop clear and relevant learning objectives for a learning module**

Category **Relevant objectives** 7

Quotes: “*Good appropriate subjects”*

*“Clear topics”*

*“Easy to grasp”*

- **Prepare reading assignments according to the objectives**

Category: **Preparation materials** 3

Quotes: “*Preparation was very good”*

*“Good tips on links and websites with useful*

*information on world health issues”*

*“Great study material and links*

*to useful websites”*

- **Prepare cases according to the objectives, including key learning points**

Category: **Relevant cases** 14

Quotes: *“Very good to work with cases from reality and*

*be able to get help from experts”*

*“Good interactive method for case presentations”*

*“Case based with good space for dialogue”*

- **Facilitate team-based case discussions**

Category: **Group discussions** 17

Quotes: “*Good concept with discussion in groups”*

*“The discussion form opens up many angles”*

*“Workshops are a very good form of learning”*

Category: **Course leaders’ skills** 14

Quotes: *“Skilful, dedicated, well-informed course leaders”*

*“Course leaders’ personality and competence”*

*“Great competence of educators and group leaders”*

Category: **Active participation** 2

Quotes: “*Everyone was involved”*

*“They activated us as participants”*

Category: **Take note of other people’s experiences** 2

Quotes: “*Participants from similar areas of work”*

*“Exchange of experiences from different units”*

Footnote: Categorisation of 10 answers to the question “*Which were the three main weaknesses of the learning module”*: one nurse thought that “*Nurses need a little more basic medical knowledge*”, another replied “*Sometimes duplication*”, one of the paediatricians stated “*Would have liked some politicians to join in to hear our discussions”*, four of the participants complained about the premises and three that the days were too long.
