# Supplementary material for: Combining learning for educators and participants in a paediatric CPD programme
Source: BMC Med Educ. 2019 Jan 21;19:28. doi: 10.1186/s12909-019-1461-x (PMC6341706; doi:10.1186/s12909-019-1461-x)
Supplement: Supplementary file 4 — Table S4. Participants’ reflections in responses to the open questions on learning and clinical practice: major theme, themes and subthemes (DOC 51 kb) [file 12909_2019_1461_MOESM4_ESM.doc]

**Table 4. Participants’ reflections in responses to the open questions on learning and clinical practice: major theme, themes and subthemes**

| **MAJOR THEME: Improvement in clinical practice** | | | |
| --- | --- | --- | --- |
| **THEMES** | **SUBTHEMES (three perspectives)** | | |
|  | **Physician/nurse** | **Professional environment** | **Child’s situation** |
| **Change in clinical practice** | Improving clinical skills | Increasing professional collaboration | Working for the best interests of the child |
|  | Greater confidence in my professional role |  |  |
|  | Expanding the area of responsibility for nurses |  |  |
|  | Improving psychological care |  |  |
| **Strengthened in clinical practice** | My clinical work | Understanding the importance of professional collaboration | The importance of seeing the child as a whole |
|  | My professional role | How we prioritise and distribute the work |  |
| **Need to learn or develop** | My clinical work | Professional collaboration | The regulations on trustee/administrator and legal representative |
|  | Workplace education |  |  |
|  | The nurse’s professional role |  |  |
| **Ways of learning or developing** | Individual study | Talk to my boss: who does what? | Collaboration between the child/family, trustee/administrator and other relevant authorities |
|  | Auscultation | Consulting colleagues |  |
|  | Workplace learning | Networking |  |
|  | Training | Teamwork |  |
|  | Courses | Co-ordination between children’s outpatient clinic and child psychiatry clinic |  |
|  | Continued search for knowledge | Collaboration |  |
|  |  | Non-prestigious discussions with colleagues |  |
