# Supplementary material for: Combining learning for educators and participants in a paediatric CPD programme
Source: BMC Med Educ. 2019 Jan 21;19:28. doi: 10.1186/s12909-019-1461-x (PMC6341706; doi:10.1186/s12909-019-1461-x)
Supplement: Supplementary file 5 — Table S5. Educators’ reflections in response to the open questions on learning and educational practice: major theme, themes and subthemes (DOC 48 kb) [file 12909_2019_1461_MOESM5_ESM.doc]

**Table 5. Educators’ reflections in response to the open questions on learning and educational practice: major theme, themes and subthemes**

| **MAJOR THEME: Using adult learning methods** | |
| --- | --- |
| **THEMES** | **SUBTHEMES** |
| **Change in educational practice** | Formulation of clear objectives |
|  | Using case discussions |
|  | Facilitating active participation |
| **Strengthened in the role of educator** | Practice according to the principles of adult learning |
|  | Taking advantage of the competence of the participant |
|  | Understanding the importance of relevance |
|  | Teaching according to participants’ needs |
|  | Having an open approach in discussions |
|  | Applying the perspective of the child |
|  | Using interactive case discussions |
|  | Understanding that teaching is an educational learning opportunity |
| **Need to learn or develop** | Teaching competence |
|  | Role as an educator |
|  | How to introduce preparatory assignments for the participants |
|  | Presentations according to adult learning principles |
|  | Active participation and discussions |
|  | Clear narrative |
|  | An open approach |
| **Ways of learning or developing** | Studying interactive learning methods |
|  | Studying teaching principles |
|  | Striving towards relevance |
|  | Feedback from colleagues and participants |
|  | Teaching as a team |
|  | Individual study |
|  | Practising |
|  | Courses |
